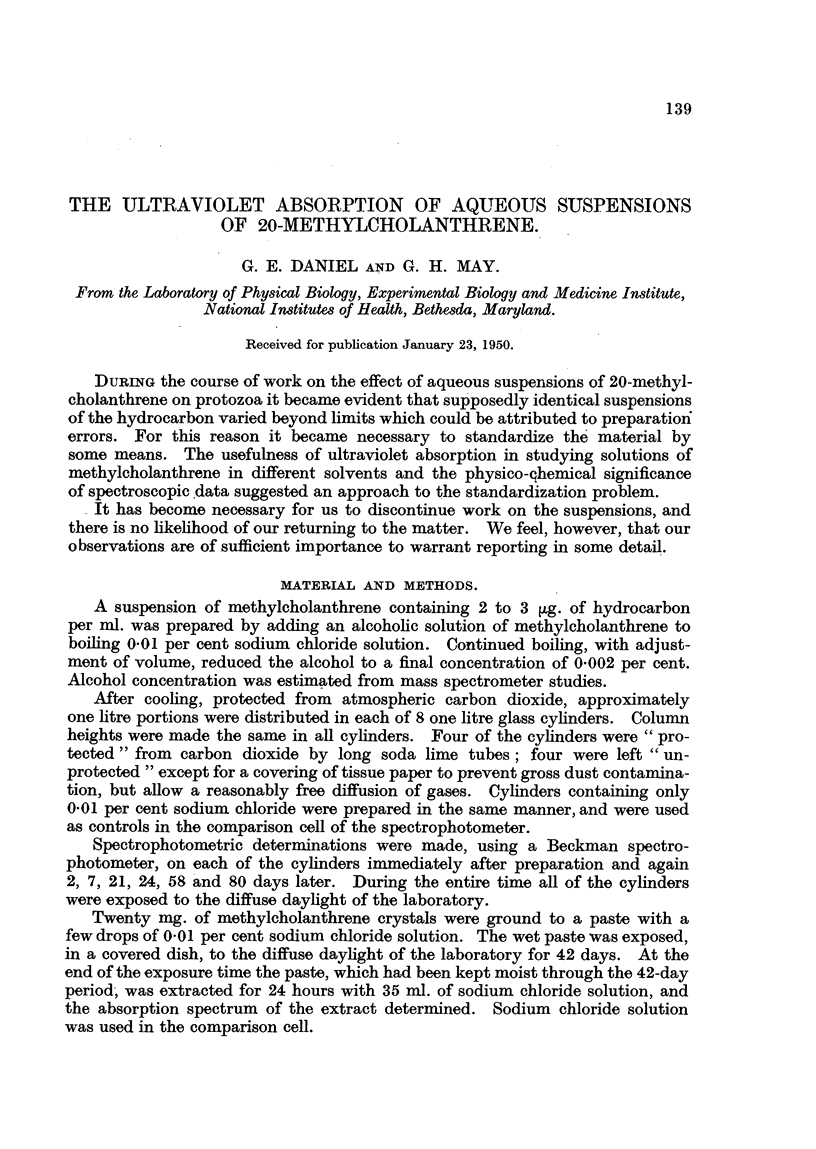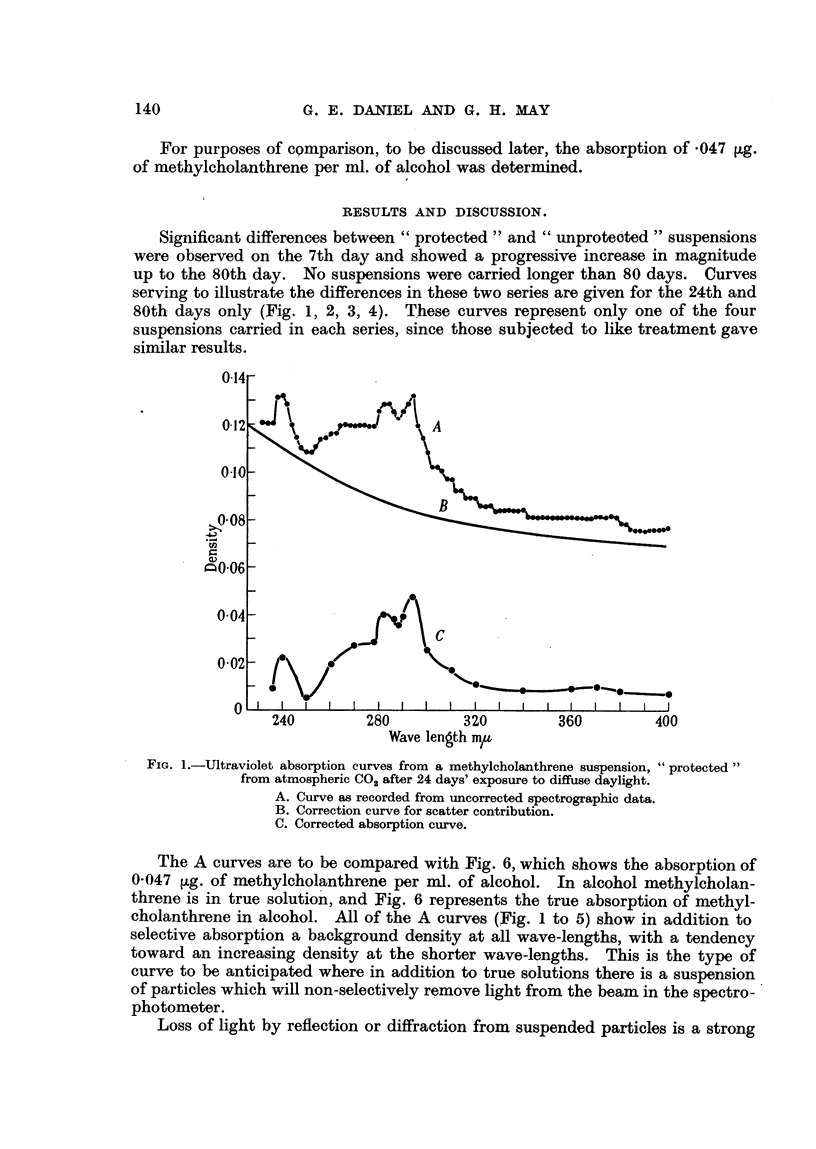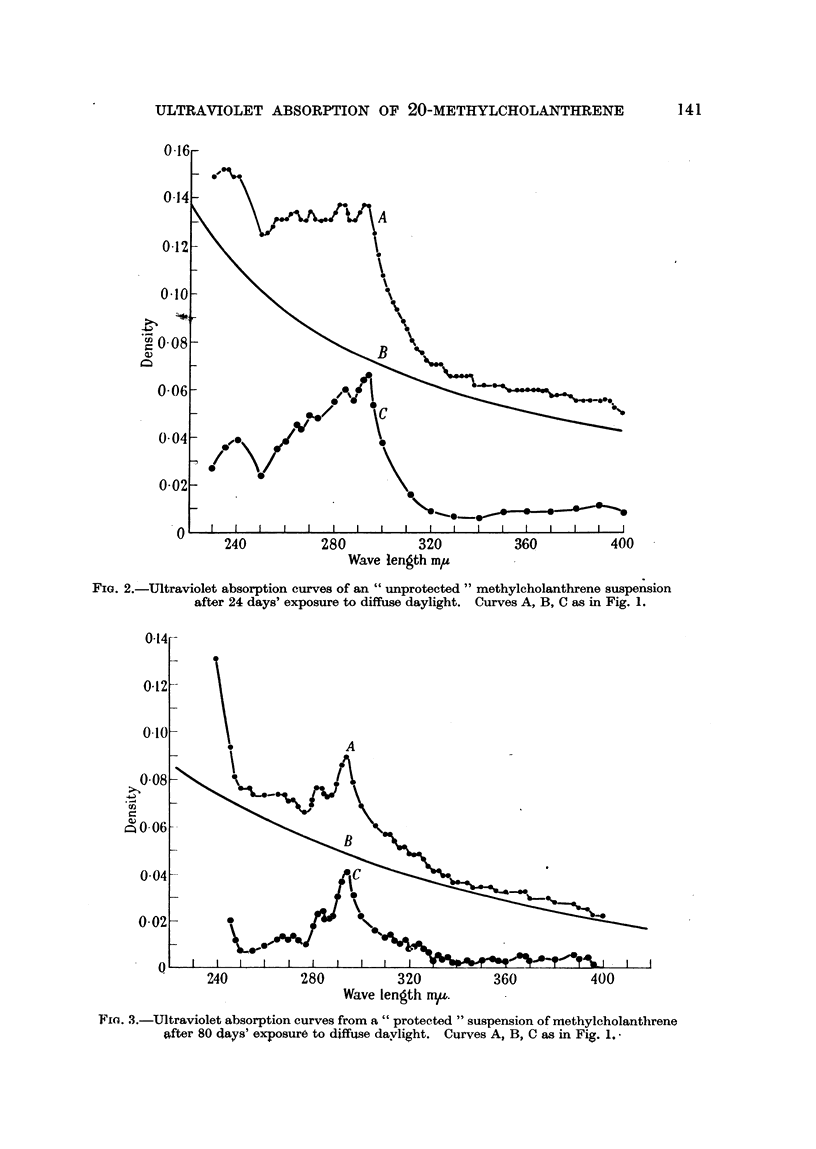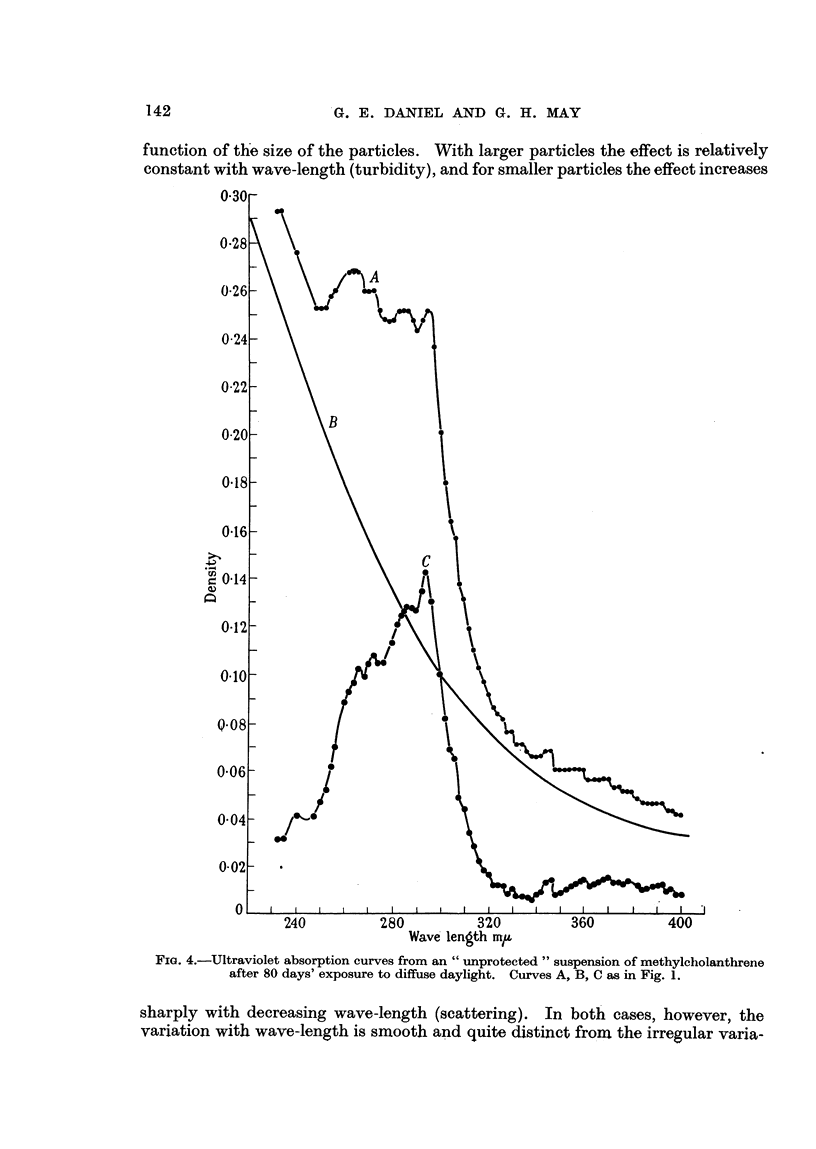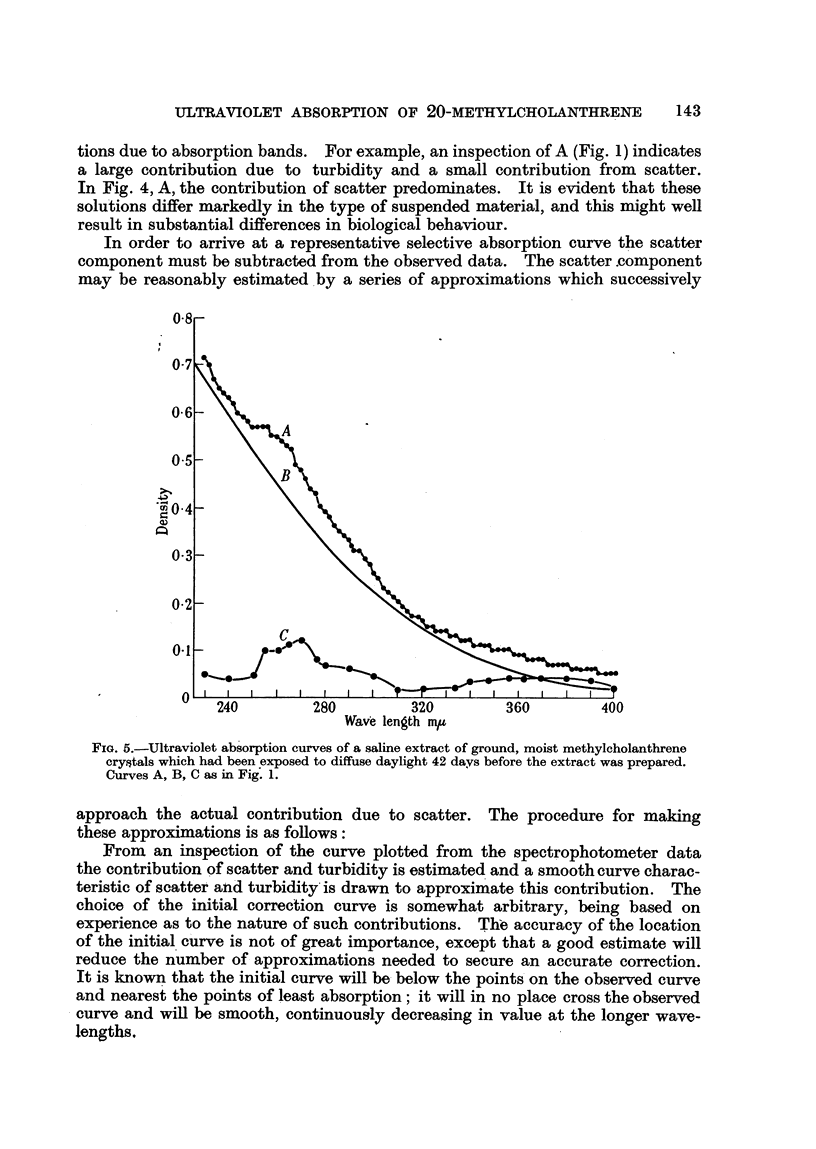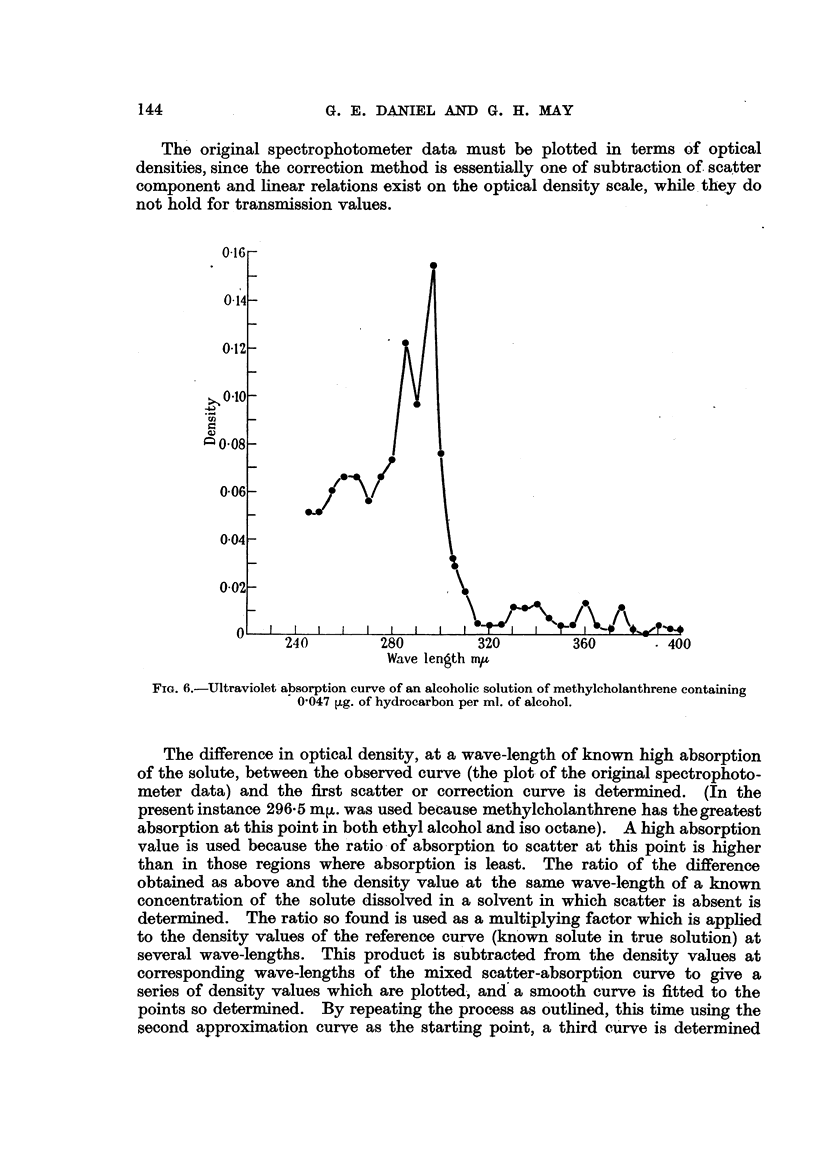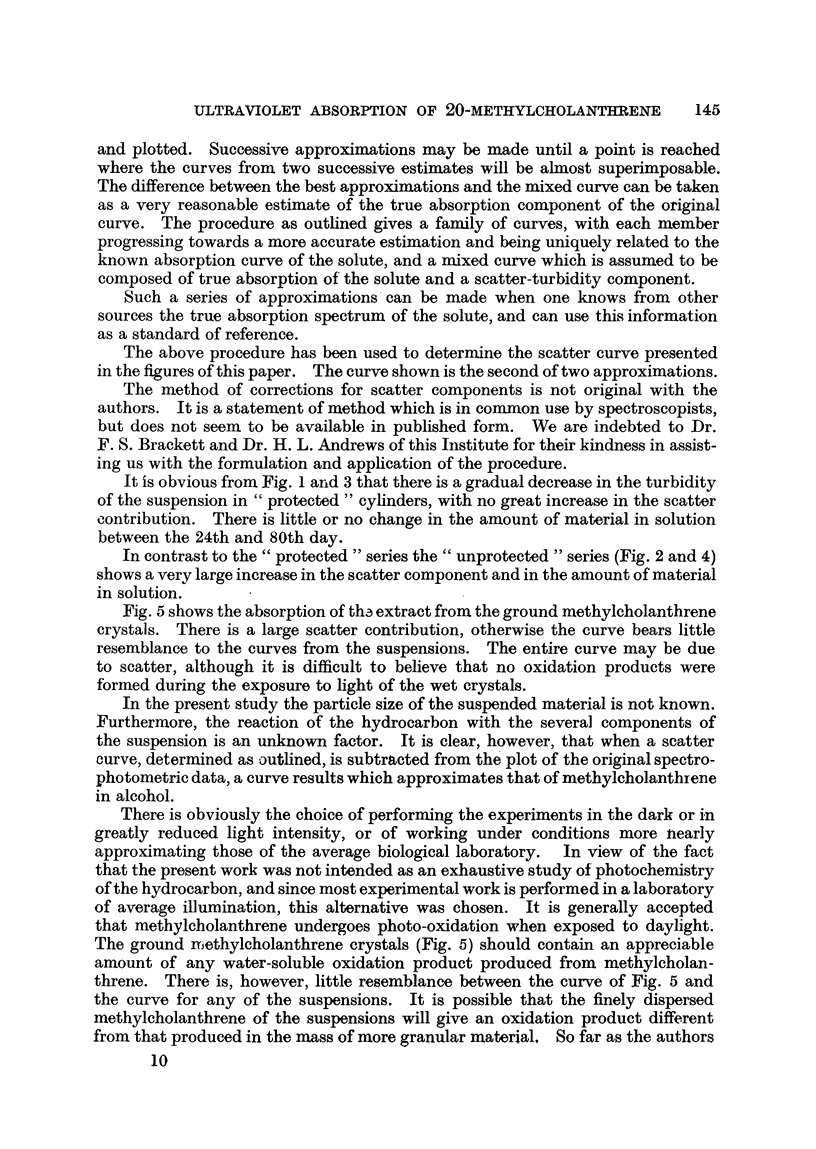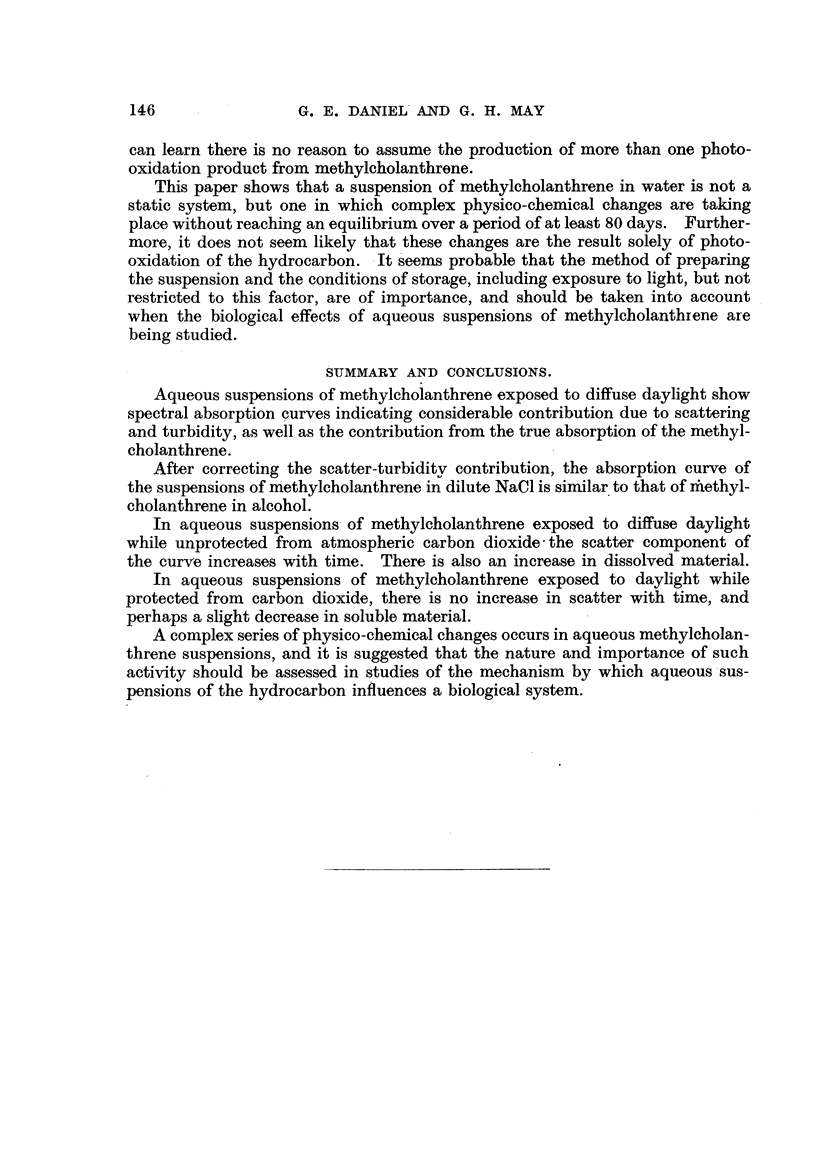# The Ultraviolet Absorption of Aqueous Suspensions of 20-Methylcholanthrene

**DOI:** 10.1038/bjc.1950.14

**Published:** 1950-03

**Authors:** G. E. Daniel, G. H. May


### THE ULTRAVIOLET ABSORPTION OF AQUEOUS SUSPENSIONS OF 20-METHYLCHOLANTHRENE.

G. E. DANIEL AND G. H. MAY.

*From the Laboratory of Physical Biology, Experimental Biology and Medicine Institute, National Institutes of Health, Bethesda, Maryland.*

Received for publication January 23, 1950.

DURING the course of work on the effect of aqueous suspensions of 20-methylcholanthrene on protozoa it became evident that supposedly identical suspensions of the hydrocarbon varied beyond limits which could be attributed to preparation errors. For this reason it became necessary to standardize the material by some means. The usefulness of ultraviolet absorption in studying solutions of methylcholanthrene in different solvents and the physico-chemical significance of spectroscopic data suggested an approach to the standardization problem.

It has become necessary for us to discontinue work on the suspensions, and there is no likelihood of our returning to the matter. We feel, however, that our observations are of sufficient importance to warrant reporting in some detail.

### MATERIAL AND METHODS.

A suspension of methylcholanthrene containing 2 to 3 $\mu$g. of hydrocarbon per ml. was prepared by adding an alcoholic solution of methylcholanthrene to boiling 0·01 per cent sodium chloride solution. Continued boiling, with adjustment of volume, reduced the alcohol to a final concentration of 0·002 per cent. Alcohol concentration was estimated from mass spectrometer studies.

After cooling, protected from atmospheric carbon dioxide, approximately one litre portions were distributed in each of 8 one litre glass cylinders. Column heights were made the same in all cylinders. Four of the cylinders were "protected" from carbon dioxide by long soda lime tubes; four were left "unprotected" except for a covering of tissue paper to prevent gross dust contamination, but allow a reasonably free diffusion of gases. Cylinders containing only 0·01 per cent sodium chloride were prepared in the same manner, and were used as controls in the comparison cell of the spectrophotometer.

Spectrophotometric determinations were made, using a Beckman spectrophotometer, on each of the cylinders immediately after preparation and again 2, 7, 21, 24, 58 and 80 days later. During the entire time all of the cylinders were exposed to the diffuse daylight of the laboratory.

Twenty mg. of methylcholanthrene crystals were ground to a paste with a few drops of 0·01 per cent sodium chloride solution. The wet paste was exposed, in a covered dish, to the diffuse daylight of the laboratory for 42 days. At the end of the exposure time the paste, which had been kept moist through the 42-day period, was extracted for 24 hours with 35 ml. of sodium chloride solution, and the absorption spectrum of the extract determined. Sodium chloride solution was used in the comparison cell.

For purposes of comparison, to be discussed later, the absorption of ·047 μg. of methylcholanthrene per ml. of alcohol was determined.

### RESULTS AND DISCUSSION.

Significant differences between " protected " and " unprotected " suspensions were observed on the 7th day and showed a progressive increase in magnitude up to the 80th day. No suspensions were carried longer than 80 days. Curves serving to illustrate the differences in these two series are given for the 24th and 80th days only (Fig. 1, 2, 3, 4). These curves represent only one of the four suspensions carried in each series, since those subjected to like treatment gave similar results.

FIG. 1.—Ultraviolet absorption curves from a methylcholanthrene suspension, " protected " from atmospheric $CO_2$ after 24 days' exposure to diffuse daylight.

A. Curve as recorded from uncorrected spectrographic data.
B. Correction curve for scatter contribution.
C. Corrected absorption curve.

The A curves are to be compared with Fig. 6, which shows the absorption of 0·047 μg. of methylcholanthrene per ml. of alcohol. In alcohol methylcholanthrene is in true solution, and Fig. 6 represents the true absorption of methylcholanthrene in alcohol. All of the A curves (Fig. 1 to 5) show in addition to selective absorption a background density at all wave-lengths, with a tendency toward an increasing density at the shorter wave-lengths. This is the type of curve to be anticipated where in addition to true solutions there is a suspension of particles which will non-selectively remove light from the beam in the spectrophotometer.

Loss of light by reflection or diffraction from suspended particles is a strong

Fig. 2.—Ultraviolet absorption curves of an " unprotected " methylcholanthrene suspension after 24 days' exposure to diffuse daylight. Curves A, B, C as in Fig. 1.

Fig. 3.—Ultraviolet absorption curves from a " protected " suspension of methylcholanthrene after 80 days' exposure to diffuse daylight. Curves A, B, C as in Fig. 1.

function of the size of the particles. With larger particles the effect is relatively constant with wave-length (turbidity), and for smaller particles the effect increases

FIG. 4.—Ultraviolet absorption curves from an " unprotected " suspension of methylcholanthrene after 80 days' exposure to diffuse daylight. Curves A, B, C as in Fig. 1.

sharply with decreasing wave-length (scattering). In both cases, however, the variation with wave-length is smooth and quite distinct from the irregular variations due to absorption bands. For example, an inspection of A (Fig. 1) indicates a large contribution due to turbidity and a small contribution from scatter. In Fig. 4, A, the contribution of scatter predominates. It is evident that these solutions differ markedly in the type of suspended material, and this might well result in substantial differences in biological behaviour.

In order to arrive at a representative selective absorption curve the scatter component must be subtracted from the observed data. The scatter component may be reasonably estimated by a series of approximations which successively

FIG. 5.—Ultraviolet absorption curves of a saline extract of ground, moist methylcholanthrene crystals which had been exposed to diffuse daylight 42 days before the extract was prepared. Curves A, B, C as in Fig. 1.

approach the actual contribution due to scatter. The procedure for making these approximations is as follows:

From an inspection of the curve plotted from the spectrophotometer data the contribution of scatter and turbidity is estimated and a smooth curve characteristic of scatter and turbidity is drawn to approximate this contribution. The choice of the initial correction curve is somewhat arbitrary, being based on experience as to the nature of such contributions. The accuracy of the location of the initial curve is not of great importance, except that a good estimate will reduce the number of approximations needed to secure an accurate correction. It is known that the initial curve will be below the points on the observed curve and nearest the points of least absorption; it will in no place cross the observed curve and will be smooth, continuously decreasing in value at the longer wavelengths.

The original spectrophotometer data must be plotted in terms of optical densities, since the correction method is essentially one of subtraction of scatter component and linear relations exist on the optical density scale, while they do not hold for transmission values.

FIG. 6.—Ultraviolet absorption curve of an alcoholic solution of methylcholanthrene containing 0·047 μg. of hydrocarbon per ml. of alcohol.

The difference in optical density, at a wave-length of known high absorption of the solute, between the observed curve (the plot of the original spectrophotometer data) and the first scatter or correction curve is determined. (In the present instance 296·5 mμ. was used because methylcholanthrene has the greatest absorption at this point in both ethyl alcohol and iso octane). A high absorption value is used because the ratio of absorption to scatter at this point is higher than in those regions where absorption is least. The ratio of the difference obtained as above and the density value at the same wave-length of a known concentration of the solute dissolved in a solvent in which scatter is absent is determined. The ratio so found is used as a multiplying factor which is applied to the density values of the reference curve (known solute in true solution) at several wave-lengths. This product is subtracted from the density values at corresponding wave-lengths of the mixed scatter-absorption curve to give a series of density values which are plotted, and a smooth curve is fitted to the points so determined. By repeating the process as outlined, this time using the second approximation curve as the starting point, a third curve is determined and plotted. Successive approximations may be made until a point is reached where the curves from two successive estimates will be almost superimposable. The difference between the best approximations and the mixed curve can be taken as a very reasonable estimate of the true absorption component of the original curve. The procedure as outlined gives a family of curves, with each member progressing towards a more accurate estimation and being uniquely related to the known absorption curve of the solute, and a mixed curve which is assumed to be composed of true absorption of the solute and a scatter-turbidity component.

Such a series of approximations can be made when one knows from other sources the true absorption spectrum of the solute, and can use this information as a standard of reference.

The above procedure has been used to determine the scatter curve presented in the figures of this paper. The curve shown is the second of two approximations.

The method of corrections for scatter components is not original with the authors. It is a statement of method which is in common use by spectroscopists, but does not seem to be available in published form. We are indebted to Dr. F. S. Brackett and Dr. H. L. Andrews of this Institute for their kindness in assisting us with the formulation and application of the procedure.

It is obvious from Fig. 1 and 3 that there is a gradual decrease in the turbidity of the suspension in " protected " cylinders, with no great increase in the scatter contribution. There is little or no change in the amount of material in solution between the 24th and 80th day.

In contrast to the " protected " series the " unprotected " series (Fig. 2 and 4) shows a very large increase in the scatter component and in the amount of material in solution.

Fig. 5 shows the absorption of the extract from the ground methylcholanthrene crystals. There is a large scatter contribution, otherwise the curve bears little resemblance to the curves from the suspensions. The entire curve may be due to scatter, although it is difficult to believe that no oxidation products were formed during the exposure to light of the wet crystals.

In the present study the particle size of the suspended material is not known. Furthermore, the reaction of the hydrocarbon with the several components of the suspension is an unknown factor. It is clear, however, that when a scatter curve, determined as outlined, is subtracted from the plot of the original spectrophotometric data, a curve results which approximates that of methylcholanthrene in alcohol.

There is obviously the choice of performing the experiments in the dark or in greatly reduced light intensity, or of working under conditions more nearly approximating those of the average biological laboratory. In view of the fact that the present work was not intended as an exhaustive study of photochemistry of the hydrocarbon, and since most experimental work is performed in a laboratory of average illumination, this alternative was chosen. It is generally accepted that methylcholanthrene undergoes photo-oxidation when exposed to daylight. The ground methylcholanthrene crystals (Fig. 5) should contain an appreciable amount of any water-soluble oxidation product produced from methylcholanthrene. There is, however, little resemblance between the curve of Fig. 5 and the curve for any of the suspensions. It is possible that the finely dispersed methylcholanthrene of the suspensions will give an oxidation product different from that produced in the mass of more granular material. So far as the authors can learn there is no reason to assume the production of more than one photo-oxidation product from methylcholanthrene.

This paper shows that a suspension of methylcholanthrene in water is not a static system, but one in which complex physico-chemical changes are taking place without reaching an equilibrium over a period of at least 80 days. Furthermore, it does not seem likely that these changes are the result solely of photo-oxidation of the hydrocarbon. It seems probable that the method of preparing the suspension and the conditions of storage, including exposure to light, but not restricted to this factor, are of importance, and should be taken into account when the biological effects of aqueous suspensions of methylcholanthrene are being studied.

### SUMMARY AND CONCLUSIONS.

Aqueous suspensions of methylcholanthrene exposed to diffuse daylight show spectral absorption curves indicating considerable contribution due to scattering and turbidity, as well as the contribution from the true absorption of the methylcholanthrene.

After correcting the scatter-turbidity contribution, the absorption curve of the suspensions of methylcholanthrene in dilute NaCl is similar to that of methylcholanthrene in alcohol.

In aqueous suspensions of methylcholanthrene exposed to diffuse daylight while unprotected from atmospheric carbon dioxide the scatter component of the curve increases with time. There is also an increase in dissolved material.

In aqueous suspensions of methylcholanthrene exposed to daylight while protected from carbon dioxide, there is no increase in scatter with time, and perhaps a slight decrease in soluble material.

A complex series of physico-chemical changes occurs in aqueous methylcholanthrene suspensions, and it is suggested that the nature and importance of such activity should be assessed in studies of the mechanism by which aqueous suspensions of the hydrocarbon influences a biological system.